# Supplementary material for: Postnatal Protein Intake as a Determinant of Skeletal Muscle Structure and Function in Mice—A Pilot Study
Source: Int J Mol Sci. 2022 Aug 8;23(15):8815. doi: 10.3390/ijms23158815 (PMC9369224; doi:10.3390/ijms23158815)
Supplement: Supplementary file 1 [file ijms-23-08815-s001.zip › ijms-1844033-supplementary.pdf]

Supplementary material

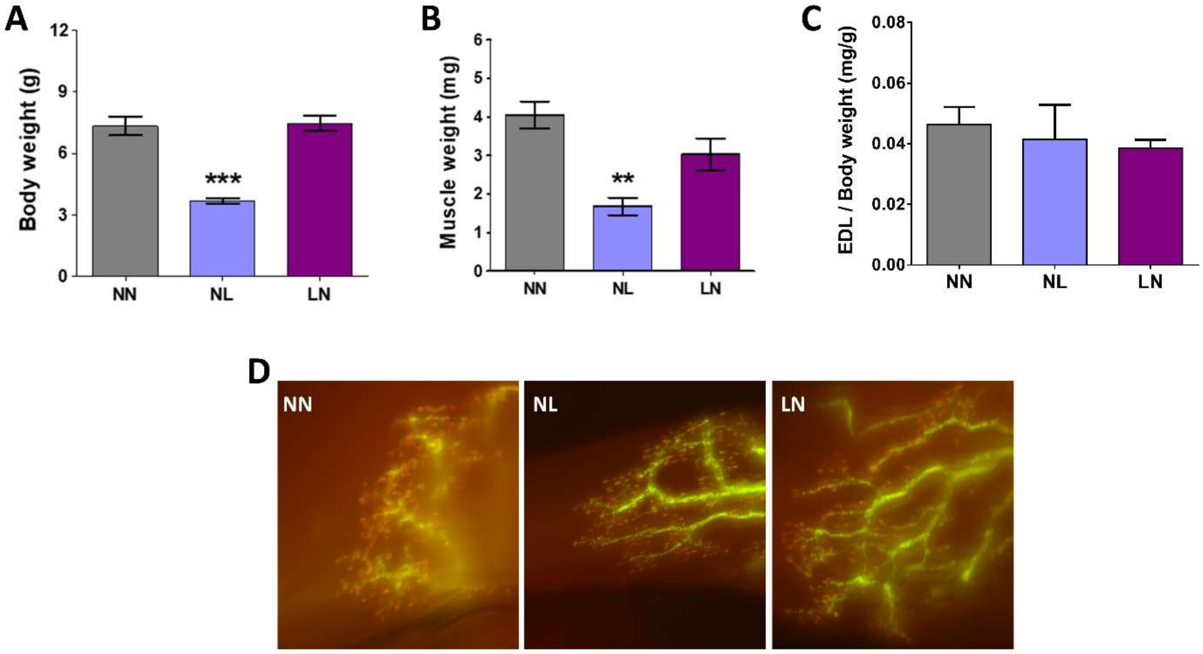

Figure S1. The effects of reduced protein intake in utero or postnatally on 21-day old mice. (**A**) Body weight, (**B**) EDL muscle weight, (**C**) EDL per total body weight ratio and (**D**) representative images of transverse sections of EDL muscle (scale bar: 100 μm, magnification: 20x), (n=11-12). NNN was used as the control group for all statistical comparisons, ***p*≤0.01, ****p* ≤0.001 (mean±SEM; One-Way ANOVA with Dunnett's post-hoc analysis).

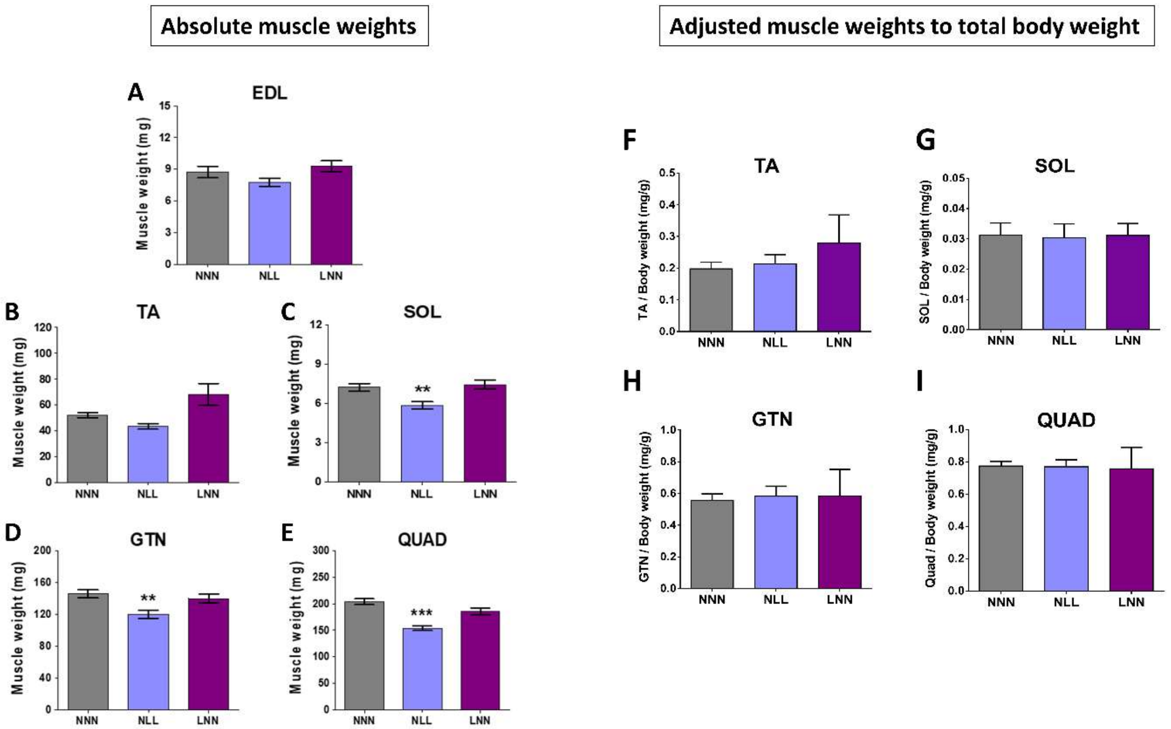

Figure S2. The effects of reduced protein intake *in utero* or postnatally on the different skeletal muscles' weight of 12-week-old mice. Absolute weights of (**A**) extensor digitorum longus (EDL), (**B**) tibialis anterior (TA), (**C**) soleus (SOL), (**D**) gastrocnemius (GTN) and (**E**) quadriceps (QUAD) are presented as well as the corresponding adjusted muscle weights to total body weight (**F**–**I**). NNN was used as the control group for all statistical comparisons, **$p \leq 0.01$, ***$p \leq 0.001$ (mean±SEM; n=5-14, One-Way ANOVA with Dunnett's post-hoc analysis).

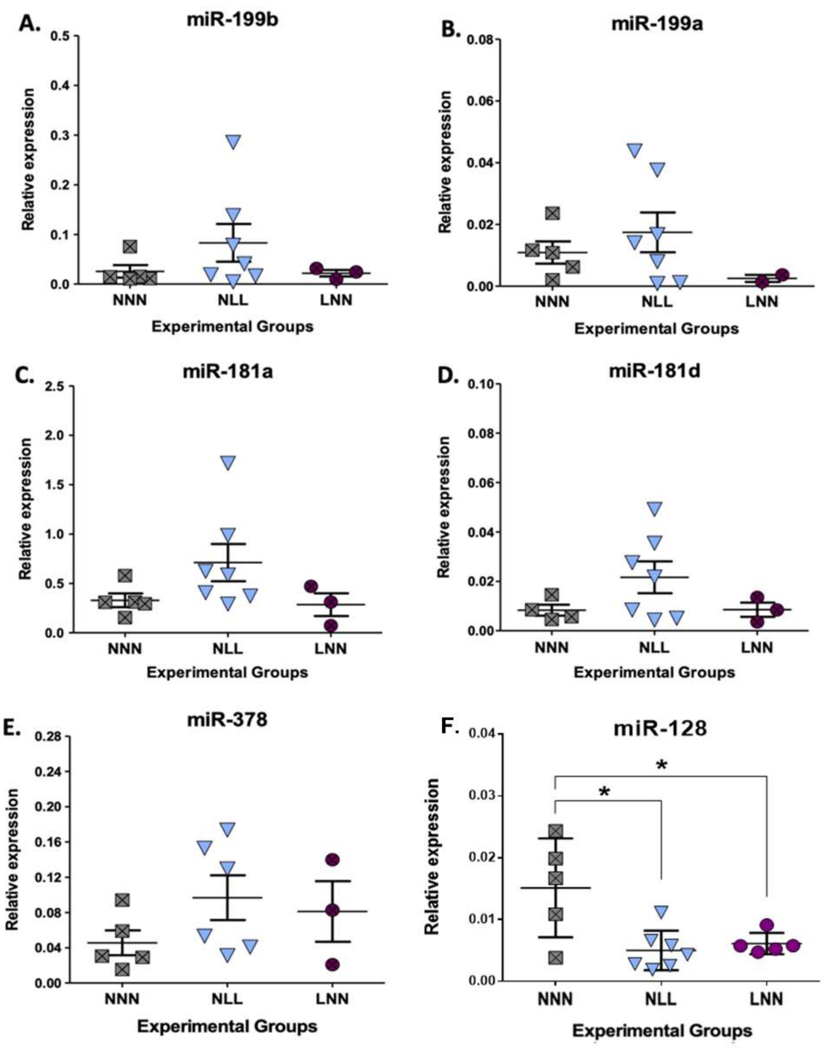

Figure S3. Expression (relative to Rnu-6) analysis of miRNAs associated with muscle development or homeostasis in TA muscle (**A**–**F**). *p≤0.05 (mean±SD; n=3-7, One-Way ANOVA with Dunnett's post-hoc analysis).

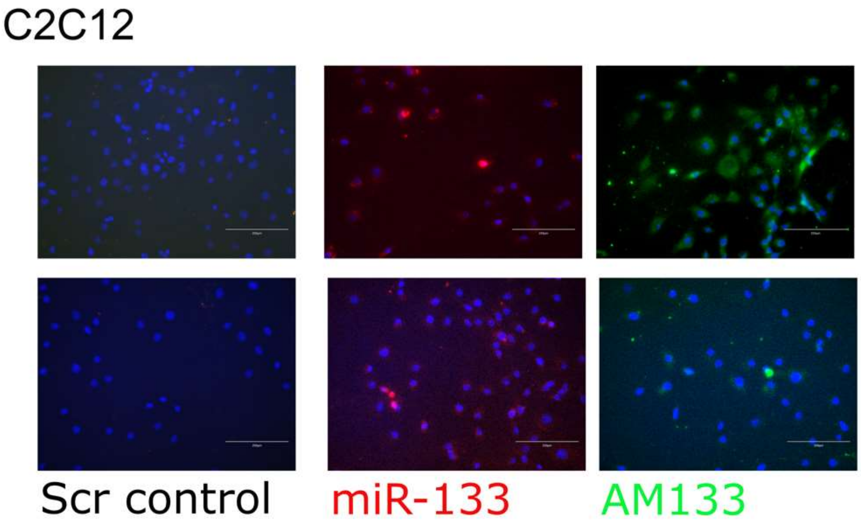

Figure S4. MiRNA tracking in C2C12 cells. C2C12 cells were treated with Cy3-conjugated miR-133 mimic or FITC-conjugated antagomiR-133 (AM133) conjugated to cholesterol for effective delivery; miR-133 and AM133 were detected in C2C12 cells 24 hours after delivery of mimic/anatgomiR. Representatvie images shown, scale bars: 250μm.

Table S1. Sequences for miR-133 scrambled control, mimic and inhibitor (antagomir, AM) used in transfections.

**miR name**		**Transfection sequence**
microRNA negative control (**Scr**)	Sense	5'mC(*)mA(*)mGmUmAmCmUmUmUmUmGmUmGmU mAmGmUmA(*)mC(*)mA(*)mA-Cholesterol-3'
	Antisense	5′-Fl-mA*mC*mUmCmAmCmCmGmAmCmAmGmC mGmUmUmGmAmA*mU*mG*mU*mU-Cholesterol-3′
miR-133a mimic (**miR-133a**)	Sense	5′-Chol-GCUGGUAAAAUGGAACCAAAU-3′
	Antisense	5′-UUUGGUCCCCUUCAACCAGCUG-3′FITC
microRNA-133a inhibitor (**AM-133a**)	Sequence	5′-Fluorescein-mC*mA*mGmCmUmGmGmUmUmGm AmAmGmGmGmGmAmCmC*mA*mA-mA*-3′Chol
	Bonds	- phsophorthioate bonds, m - 2′-O-methyl bonds

Table S2. Primer list used for qPCR of selected genes.

**Gene name**	**Primer sequences (5′ – 3′)**	
	Forward	Reverse
**Atrogin-1**	GCAGAGAGTCGGCAAGTC	CAGGTCGGTGATCGTGAG
**FoxO-3**	AGTGGATGGTGCGCTGTGT	CTGTGCAGGGACAGGTTGT
**MuSK**	GCCTTCAGCGGGACTGAG	GAGGCGTGGTGACAGG
**MyHC I**	TTGTGCCGTAGGAATGTGGG	CCTTTCTCGGAGCCACCTTG
**MyHC IIa**	CTCCAAGGACCCTCTTATTTCCC	ACTGCTGAACTCACAGACCC
**MyHC IIb**	GAGGCAATCAGGAACCTTCGG	TGTGTGTCCTTCAGCATTCCC
**MyHC IId/x**	AAGTTTGGACCCACGGTCG	CAGTGAGAGAGCCTGCCTTTA
**18S**	CGGCTACCACATCCAAGGAAGG	CCCGCTCCCAAGATCCAACTAC

Table S3. MiRNA primer list used for qPCR measurements.

Mature miR ID	miRbase Accession No	Sequence	miScript Primer Assay Catalog #
mmu-miR-199a-5p	MIMAT0000229	CCCAGUGUUCAGACUACCUGUUC	MS00007889
mmu-miR-199b-5p	MIMAT0000672	CCCAGUGUUUAGACUACCUGUUC	MS00032536
mmu-miR-181a-5p	MIMAT0000210	AACAUUCAACGCUGUCGGUGAGU	MS00011263
mmu-miR-181d-5p	MIMAT0004324	AACAUUCAUUGUUGUCGGUGGGU	MS00011284
mmu-miR-378a-5p	MIMAT0000742	CUCCUGACUCCAGGUCCUGUGU	MS00032788
mmu-miR-128-3p	MIMAT0016982	CGGGGCCGUAGCACUGUCUGA	MS00011116
SNORD-61	ENSG00000206979	-	MS00033705
